# Eliminating the need of serum testing using low serum culture conditions for human bone marrow-derived mesenchymal stromal cell expansion

**DOI:** 10.1186/1475-925X-12-15

**Published:** 2013-02-20

**Authors:** Jessica Wappler, Björn Rath, Tanja Läufer, Axel Heidenreich, Katrin Montzka

**Affiliations:** 1 Department of Urology, RWTH Aachen University, Pauwelsstraße 30, 52074, Aachen, Germany; 2 Department of Orthopaedic Surgery, RWTH Aachen University, Pauwelsstraße 30, 52074, Aachen, Germany

**Keywords:** Cytotoxicity, Mesenchymal stromal cells, MSC-tested serum, Proliferation, Serum-reduced medium

## Abstract

**Background:**

The conventional expansion of human mesenchymal stromal cells (hMSC) for tissue engineering or (pre-) clinical investigation includes the use of 10% fetal bovine serum (FBS). However, there exists immense lot-to-lot variability in FBS samples and time consuming as well as cost intensive lot pre-testing is essential to guarantee optimal hMSC proliferation and stem cells characteristics maintenance. Furthermore, lot-to-lot variability impedes the long-term consistency of research and comparability between research groups. Therefore, we investigated the use of defined, invariable, non-synthetic FBS in low serum culture conditions for isolation and expansion of hMSC.

**Methods:**

hMSC were isolated from bone marrow in Panserin 401 supplemented with growth factors and 2% MSC-tested or non-tested, defined, invariable, non-synthetic FBS and further cultivated *in vitro*. The surface marker expression, differentiation capacity as well as cell proliferation and cytotoxicity was analyzed and compared between serum samples.

**Results:**

Cells isolated and cultivated with low concentrations of MSC-tested or non-tested FBS demonstrated no differences in surface marker expression or differentiation capacity. Proliferation of hMSC was equal in medium supplemented with either serum with no indication of cell death.

**Conclusions:**

The low serum concentration in Panserin 401 supplemented with growth factors enables the use of defined, invariable, non-synthetic FBS for the isolation and expansion of hMSC. The required hMSC characteristics like surface marker expression and differentiation capacity are maintained. Importantly, no differences in the cell proliferation could be detected. Therefore, using these low-serum culture conditions, the need for lot-to-lot pre-testing of FBS usually needed for optimal hMSC expansion is abolished leading to long-term consistency and comparability of results.

## Background

The therapeutic application of human mesenchymal stromal cells (hMSC) is of great interest in a variety of diseases including graft-versus-host disease (GvHD) [1,2], osteogenesis imperfecta [3,4], acute myocardial infarction [5,6], spinal cord injury [7,8], multiple sclerosis [9,10], and critical limb ischemia [11,12]. Although hMSC can differentiate into adipocytes, osteocytes and chondrocytes [13], the beneficial effect in the above mentioned diseases is a paracrine mechanism [14]. hMSC are capable of expressing and releasing a variety of different growth factors (basic fibroblast growth factor (bFGF), epidermal growth factor (EGF), hepatocyte growth factor (HGF), platelet-derived growth factor (PDGF), stromal-derived growth factor 1α (SDF-1 α), transforming growth factor β (TGF-β), vascular endothelial growth factor (VEGF)) and cytokines (interleukin 1 β (IL-1β), IL-6, IL-8, tumor necrosis factor α (TNFα)) in a donor-dependent manner [15-19].

For tissue engineering purposes as well as for the clinical application of hMSC, adequate numbers of viable, transplantable (autologous or allogenic) cells need to be generated. Since hMSC represent only a small proportion (0.01-0.02%) of bone marrow cells, the expansion to high cell numbers is crucial [20]. The conventional applied medium for such expansion procedures is Dulbecco’s modified Eagle Medium (DMEM) supplemented with 10% fetal bovine serum (FBS) [21]. FBS has, however, the disadvantage that there exists immense lot-to-lot variability and unexpected cell growth characteristics [22]. To overcome these difficulties, time-consuming lot pre-testing is required to guarantee optimal cell growth and maintenance of hMSC characteristics. Alternatively, the use of commercial hMSC-tested serum can be applied. However, these pre-tested sera are highly expensive and lot-to-lot variability still exists. In a previous study, we were able to develop serum-reduced culture conditions for even better hMSC proliferation than conventional DMEM with 10% FBS [23]. This serum-reduced medium consists of Panserin 401 supplemented with only 2% FBS and growth factors. The aim of the current study was to evaluate if this low serum concentration can be replaced by defined, invariable, non-synthetic FBS which is available at around 10% of the costs of commercially pre-tested sera and guarantees the consistency and comparability of research results.

## Methods

### Cell culture

Cancellous bone fragments were obtained during operation procedures from four fully anonymous patients (with informed consent according to local ethical board approval of the University Hospital, Aachen) and selected by their plastic adherence as demonstrated previously [15,17,23]. Briefly, bone fragments were washed with Panserin 401 media (Pan Biotech GmbH, Aidenbach, Germany) supplemented with 2% FBS (hMSC-tested serum: PromoCell, Heidelberg, Germany; non-tested serum: FBS Gold, PAA Laboratories GmbH, Pasching, Austria) and growth factors (10 ng/ml EGF, 1 ng/ml bFGF, 1 ng/ml PDGF-BB; all PeproTech GmbH, Hamburg, Germany) and 10 nM dexamathasone (Sigma, Steinheim, Germany). The collected media was centrifuged with 500 g for 5 min and the cell pellet was resuspended in 10 ml fresh media, transferred to a T75 flask (Greiner Bio-One, Frickenhausen, Germany) and maintained in a humidified cell culture incubator at 37°C and 5% CO_2_. Non-adherent cells were removed by media exchange on the next day. Medium exchange was performed every 3–4 days. Pictures were taken using a Leica DMI4000 B microscope (Leica, Wetzlar, Germany) and Diskus software (Carl H. Hilgers Technisches Büro, Königswinter, Germany). When the cells reached nearly confluence, they were detached with trypsin/ethylene diamine tetra acetic acid (EDTA) (PAA) and re-seeded with a density of 4000 cells/cm^2^. All experiments were performed with cells of passage 1.

### hMSC characterization

Surface marker profiles and multipotent differentiation capacities of isolated hMSC of those four donors were analyzed according to the guidelines set by the *Mesenchymal and Tissue Stem Cell Committee of the International Society for Cellular Therapy*[13] as demonstrated previously [23,24].

To detect specific surface antigens, cells were detached and fixed with 4% paraformaldehyde for 20 min. After washing with 0.1 M phosphate-buffered saline (PBS), cells were incubated in blocking solution (20% FBS in PBS) for 20 min. After washing with PBS, the cell pellets (300,000 per antibody) were resuspended in 100 μl PBS, 2 μl primary antibody was added and incubated for 30 min. Monoclonal primary antibodies recognizing surface markers CD11b (Invitrogen, Carlsbad, USA), CD19, CD34, CD45, CD73, CD90 (Becton Dickinson, San Jose, USA), CD105 (Invitrogen) and HLA-DR (Abcam, Cambridge, UK) were used. After three washing steps with PBS, 2 μl of the secondary antibody (Alexa-488 conjugated goat anti-mouse, Invitrogen) was incubated in 100 μl PBS for 30 min in the dark. After two final washing steps, the cells were re-suspended in 400 μl PBS and analyzed using a FACSCalibur and FACSCalibur software (Becton Dickinson, Heidelberg, Germany).

For adipogenic differentiation, hMSC were cultivated alternately in adipogenic induction medium and adipogenic maintenance medium for 3–4 days for 3 weeks. Adipogenic induction medium consisted of DMEM (Lonza, Verviers, Belgium) containing 10% FBS, 0.01 mg/ml insulin, 0.2 μM indomethacin, and 0.5 mM isobutyl-methyl-xanthine (all Sigma). Adipogenic maintanance medium consisted of DMEM supplemented with 10% FBS and 0.01 mg/ml insulin. Oil Red O staining (Sigma) was used to visualize lipid droplets formation in differentiated adipocytes. Osteogenic differentiation was induced by cultivation of hMSC in DMEM containing 10% FCS, 10 nM sodium β-glycerophosphate, 100 nM dexamethasone, and 0.05 mM 1-ascorbic acid 2-phosphate (all Sigma). Medium was changed every 3 days for 3 weeks. Osteogenic differentiation was visualized by Alizarin Red S (Sigma) staining to demonstrate matrix mineralization associated with osteoblast differentiation.

### Proliferation and cytotoxicity

Cell proliferation and cytotoxicity were measured using the CellTiter-Blue Cell Viability Assay and CytoTox-ONE Homogeneous Membrane Integrity Assay (Promega, Madison, USA) according to the manufacturer’s protocol twice a week for two weeks without passaging of cells [23]. Therefore, 5 × 10^4^ cells of four donors were seeded into 12-wells and grown in culture media supplemented with the different sera. For cytotoxicity measurement, 100 μl of the supernatant were transferred into a black 96-well plate, and incubated with 100 μl CytoTox-ONE for 10 min at 37°C. To measure proliferation, CellTiter-Blue was diluted 1:5 in medium and 1 ml added to each well for 1 h at 37°C and 5% CO_2._ Subsequently, 200 μl of the supernatant were transferred into a black 96-well plate. For both assays fluorescence intensity was measured using the fluorometer FLUOstar OPTIMA (BMG Labtech, Jena, Germany) with an excitation at 560 nm and emission at 590 nm. After washing with medium, 1 ml fresh medium was added to the cells and incubated until the following measurement.

### Statistical analysis

All data are presented as the mean value ± SD. Statistical differences were determined by one-way ANOVA followed by Bonferroni post hoc testing. A p value of less than 0.05 was considered to be significant. All the statistical analyses and the evaluation of data were performed using GraphPad Prism version 4.03 (GraphPad Software, San Diego, USA).

## Results

To evaluate if serum-reduced culture conditions for expansion of bone marrow-derived hMSC to relevant cell numbers for tissue engineering purposes or direct clinical application might be performed with defined, invariable, non-synthetic FBS rather than MSC-tested FBS, hMSC of four different donors were isolated in Panserin 401 supplemented with growth factors and 2% MSC- or non-tested serum. Cultivated hMSC were characterized according to the *Mesenchymal Stem Cell Committee of the International Society for Cellular Therapy*[13] by three criteria: (I) adherence to tissue culture plastic, (II) specific surface marker expression, and (III) multipotent differentiation capacity.

**Figure 1:**
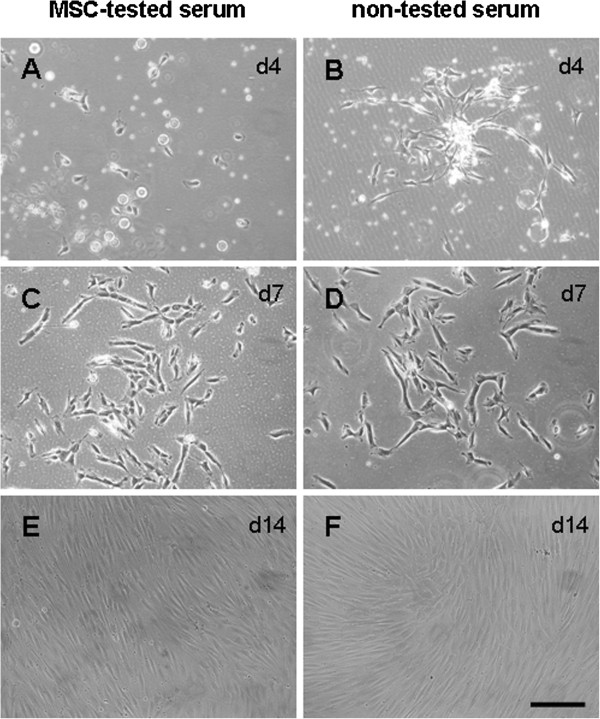
**Isolation of hMSC with MSC-tested or non-tested serum resulted in similar cell morphology and cell growth.** Cell growth of isolated hMSC was monitored after d4 (**A** + **B**), d7 (**C** + **D**) and d14 (**E** + **F**). Growth of hMSC in either serum resulted in confluent cultures after 14d. Scale: 200 μm.

Isolation of hMSC was performed simultaneously in Panserin 401 medium supplemented with 2% MSC-tested or non-tested serum (Figure 1A + B). Cell growth was monitored after 7 (Figure 1C + D) and 14 days (Figure 1E + F). After 14 days in culture, hMSC grown with either serum demonstrated confluent cultures which were further processed for surface marker and differentiation capacity analysis.

**Figure 2:**
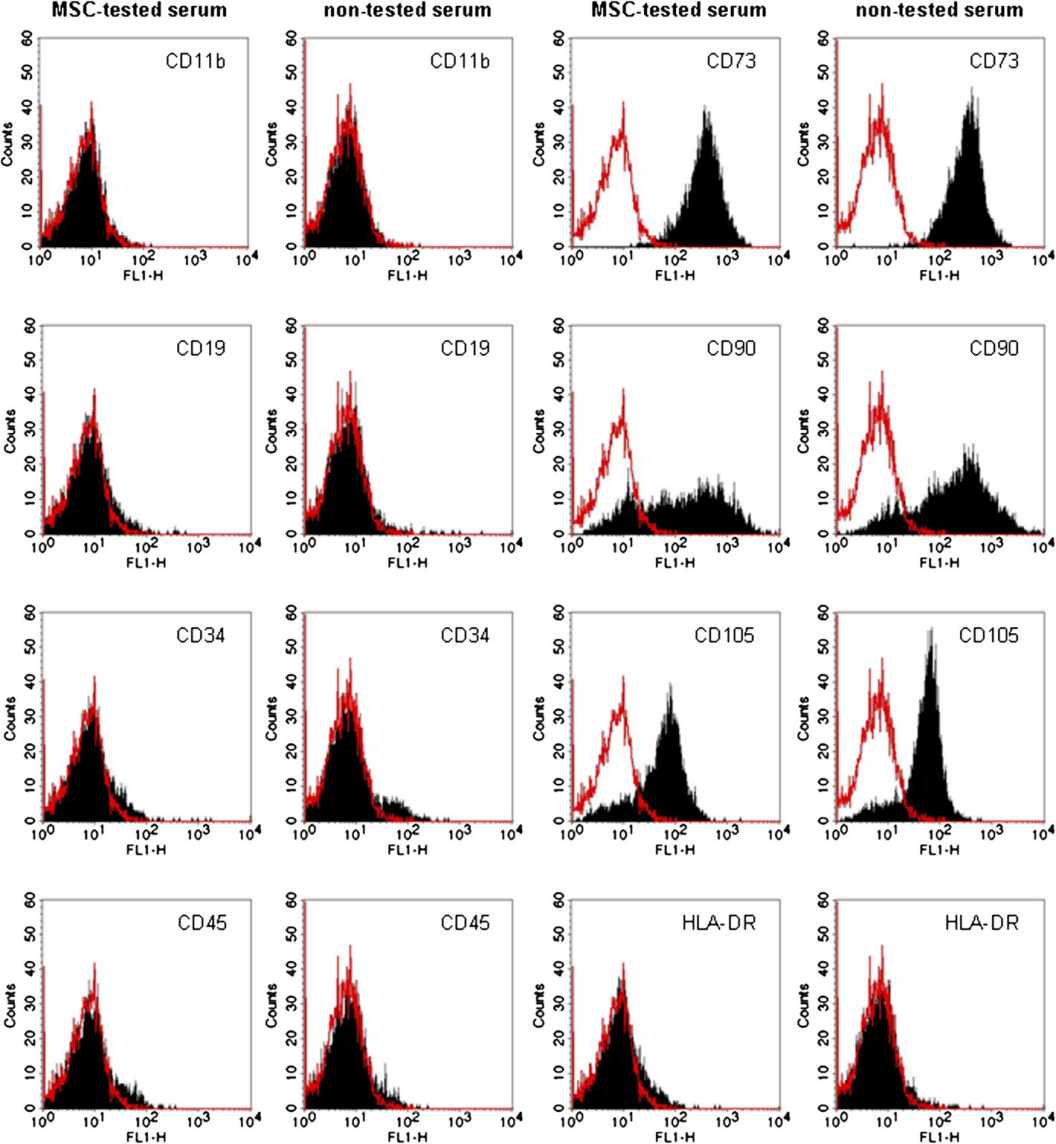
**No alteration of hMSC surface marker expression after isolation and cultivation with MSC-tested or non-tested serum.** FACS analysis of the immunophenotypic surface profile for CD11b, CD19, CD34, CD45, CD73, CD90, CD105 and HLA-DR show no differences between hMSC isolated and expanded with MSC-tested or non-tested FBS. Red histograms represent the fluorescence from negative control cells incubated with only secondary antibody.

**Figure 3:**
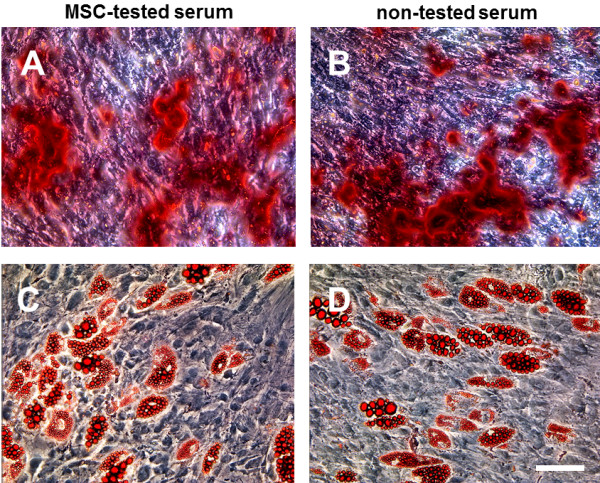
**Differentiation capacity of hMSC is not altered by cultivation with MSC-tested or non-tested serum.** hMSC isolated and cultivated with MSC-tested or non-tested FBS were differentiated using osteogenic or adipogenic induction protocols for three weeks. Osteogenic differentiation (**A** + **B**) was demonstrated by Alician Red S staining of mineral depositions and adipogenic differentiation (**C** + **D**) by Oil Red O staining of lipid droplets. Scale: 100 μm.

Fluorescent activated cell sorting (FACS) analysis of adherent hMSC isolated and expanded with both sera demonstrated the expression of the surface markers CD73, CD90 and CD105, as well as the absence of CD11b, CD19, CD34, CD45 and HLA-DR (Figure 2). To investigate the multipotent differentiation potential of hMSC isolated and cultivated with both sera, hMSC were differentiated into adipocytes and osteocytes. Alizarin Red S staining was performed to detect mineral deposition after osteogenic differentiation (Figure 3A + B). No staining differences between the different sera could be identified. Oil Red O staining was applied to visualize the lipid vacuoles in differentiated adipocytes (Figure 3C + D). No differences in the degree of lipid vacuole production could be detected between the employed sera. 

**Figure 4:**
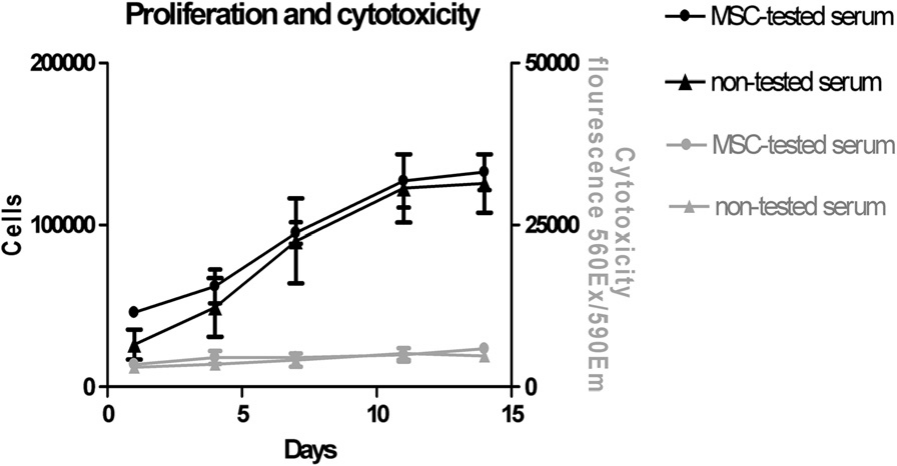
**Cell proliferation of hMSC is not altered by cultivation with MSC-tested or non-tested serum.** Cell Proliferation and cytotoxicity was monitored every 3–4 days using CellTiter-Blue Cell Viability Assay and CytoTox-ONE Homogeneous Membrane Integrity Assay. No significant differences in cell proliferation could be observed between hMSC isolated and cultivated in MSC-tested or non-tested FBS (black graph). None of the applied sera induced cell death (grey graph). Data are expressed as mean values ± SD.

Besides the maintenance of hMSC characteristics by applied culture conditions, the growth promoting properties of the individual serum is of great importance. Therefore, hMSC isolated and grown in both sera were evaluated regarding their proliferative capacity. The day after seeding 5 × 10^4^ cells into each well, the measurement of day 1 revealed that hMSC that were cultivated in non-tested serum demonstrated lower cell attachment than hMSC that were cultivated in MSC-tested serum (Figure 4). However, this difference was not significant (MSC-tested serum: 4.6 ± 0.6 × 10^4^ cells; non-tested serum: 2.6 ± 0.9 × 10^4^ cells). Cells of all four donors proliferated in both sera and hMSC grown with non-tested serum reached equal cell numbers after 7 days in culture (MSC-tested serum: 9.5 ± 1.3 × 10^4^ cells; non-tested serum: 9.0 ± 2.6 × 10^4^ cells). At the end of the experiment at 14 days after seeding, hMSC grown in both sera demonstrated confluent cell cultures (MSC-tested serum: 13.3 ± 1.1 × 10^4^ cells; non-tested serum: 12.6 ± 1.8 × 10^4^ cells). Furthermore, evaluation of the cytotoxicity was performed to detect cell death. Neither of the applied sera demonstrated any cytotoxicity to cultivated hMSC over the whole experimental procedure.

## Discussion

The use of pre-tested sera is essential to guarantee optimal hMSC proliferation and maintenance of hMSC characteristics. The aim of the current study was to investigate if in low serum culture conditions MSC-tested serum might be exchanged by defined, invariable, non-synthetic FBS. Therefore, hMSC of four different donors were isolated and expanded in both sera and compared regarding their surface marker expression, differentiation capacity and cell proliferation. Analysis of the surface marker expression demonstrated the existence of CD73, CD90 and CD105 as well as the absence of CD11b, CD19, CD34, CD45 and HLA-DR of hMSC expanded with either serum. Furthermore, the differentiation capacity into adipocytes and osteocytes was not affected by the different sera. According to the *Mesenchymal Stem Cell Committee of the International Society for Cellular Therapy *[13], all cells isolated by media supplemented with MSC-tested or non-tested serum were proved to be hMSC.

Important for tissue engineering purposes as well as for the clinical application of isolated hMSC is, however, the generation of relevant cell numbers. In a previous study, we were able to demonstrate higher cell proliferation using low serum culture conditions based on Panserin 401 than the conventional applied DMEM with 10% FBS [23]. Comparison of the hMSC proliferation in Panserin 401 supplemented with growth factors and 2% of MSC-tested or non-tested serum revealed no significant differences. Interestingly, lower cell attachment could be detected in cultures that were cultivated with non-tested serum. However, this difference was not significant and could be explained by discrepancies in attachment factor concentration which might also exist between different MSC-tested lots. Since hMSC cultivated with non-tested serum reached equal cell numbers already after 7 days in culture, this lower attachment capacity can be disregarded. In conclusion, hMSC isolated and cultivated in Panserin 401 supplemented with growth factors and 2% non-tested FBS maintain their cell-specific characteristics and proliferative capacity.

The defined, invariable, non-synthetic FBS used in this study is chromatographically purified and fractionated. Afterwards, the individual constituents are recombined to guarantee the defined composition (http://www.paa.com). The advantage of the use of this FBS is, besides the lower price, that the necessity of serum lot testing is abolished. Furthermore, research groups from all over the world could work with the same FBS which might lead to more comparable results as well as an efficient transfer of results to the clinic.

In an attempt to eliminate FBS in hMSC culture conditions, Lohmann et al. [25] investigated the use of human platelet lysate (HPL). Primary isolation and differentiation by using 10% HPL could be achieved; however, the HPL donor age immensely affected hMSC proliferation rate.

Other groups (see Table 1) attempt to totally eliminate FBS or human serum components in hMSC cultivation since serum always carries the risk for the transmission of infectious agents and the potential for promoting or enhancing immune rejection [26,27]. The approach by Mimura et al. is based on a medium that was initially developed for embryonic stem cell cultivation [25] and further supplemented with bFGF, heparin and TGF-β1 [26]. In their investigation, they cultivated an hMSC cell line (UE7T-13) and demonstrated the maintenance of CD73, CD90 and CD105 expression as well as adipogenic and osteogenic differentiation capacity. However, these results were obtained with a single hMSC cell line and the capacity to efficiently isolate hMSC from bone marrow using this serum-free culture medium still needs to be demonstrated. Chase and colleagues employed a commercially available medium (StemPro MSC SFM by Invitrogen) to investigate hMSC proliferation [27]. Although this serum-free medium revealed the same proliferation rate than the conventional DMEM with 10% FBS, the differentiation capacity was only presented in samples that were isolated with serum-containing medium. Furthermore, the manufacturer himself only demonstrated the tri-lineage differentiation capacity of hMSC grown in this special serum-free culture medium (http://www.invitrogen.com). The differentiation capacity of cells that were isolated in this medium is still lacking. Although these chemically-defined media are thought to be safer and therefore better for clinical settings, the propriety composition of these hMSC culture media might impede the clinical acceptance. Even though the use of FBS in hMSC culture media is of concern, hMSC isolated and cultivated in FBS-containing medium have been approved by the US Food and Drug Administration (FDA) for use in a variety of clinical trials (http://clinicaltrials.gov).

**Table 1:** Main cultivation protocols of human bone marrow-derived MSC (grey: serum-free protocol)

**Medium**	**Reference**	**Primary isolation demonstrated**	**Differentiation demonstrated**
DMEM 10% FBS	Chase et al. [27], Lohmann et al. [28], Montzka et al. [23]	yes	yes
DMEM/F12 20% FBS or 20% human autologous serum or 20% human allogenic serum	Shahdadfar et al. [21]	yes	yes
DMEM 10% HPLs	Lohmann et al. [28]	yes	yes
Panserin 401 2% FBS and GF	Montzka et al. [23]	yes	yes
ESF basal medium and supplements	Mimura et. al [26]	no	yes
Stem Pro MSC SFM (Invitrogen)	Chase et al. [27]	no	yes

## Conclusions

In conclusion, the current study demonstrated the isolation, expansion and maintenance of hMSC characteristics by the use of low serum concentrations using a defined, invariable, non-synthetic FBS. This media composition enables the generation of consistent research results over long periods and importantly, simplifies the collaboration and comparability within different research groups.

## Abbreviations

bFGF: Basic fibroblast growth factor; DMEM: Dulbecco’s modified eagle medium; EDTA: Ethylene diamine tetra acetic acid; EGF: Epidermal growth factor; FBS: Fetal bovine serum; FDA: US food and drug administration; GvHD: Graft-versus-host disease; HGF: Hepatocyte growth factor; hMSC: Human mesenchymal stromal cells; HPL: Human platelet lysate; IL: Interleukin; PBS: Phosphate-buffered saline; PDGF: Platelet-derived growth factor; SDF: Stromal-derived factor; TGF: Transforming growth factor; TNF: Tumor necrosis factor; VEGF: Vascular endothelial growth factor.

## Competing interests

The authors declare that they have no competing interests.

## Authors’ contributions

JW isolated the hMSC, performed the cell culture experiments and was involved in drafting the manuscript. BR participated in the design of the study and performed the bone surgeries for the hMSC isolation. TL participated in the design of the study and was involved in the staining of differentiated hMSC. AH was involved in the conceiving of the study and helped to draft the manuscript. KM conceived, designed and coordinated the study and wrote the manuscript. All authors read and approved the final manuscript.
